# Supplementary material for: Gut colonisation with multidrug-resistant Klebsiella pneumoniae worsens Pseudomonas aeruginosa lung infection
Source: Nat Commun. 2023 Jan 5;14:78. doi: 10.1038/s41467-022-35767-4 (PMC9816093; doi:10.1038/s41467-022-35767-4)
Supplement: Supplementary file 1 — Supplementary Information [file 41467_2022_35767_MOESM1_ESM.pdf]

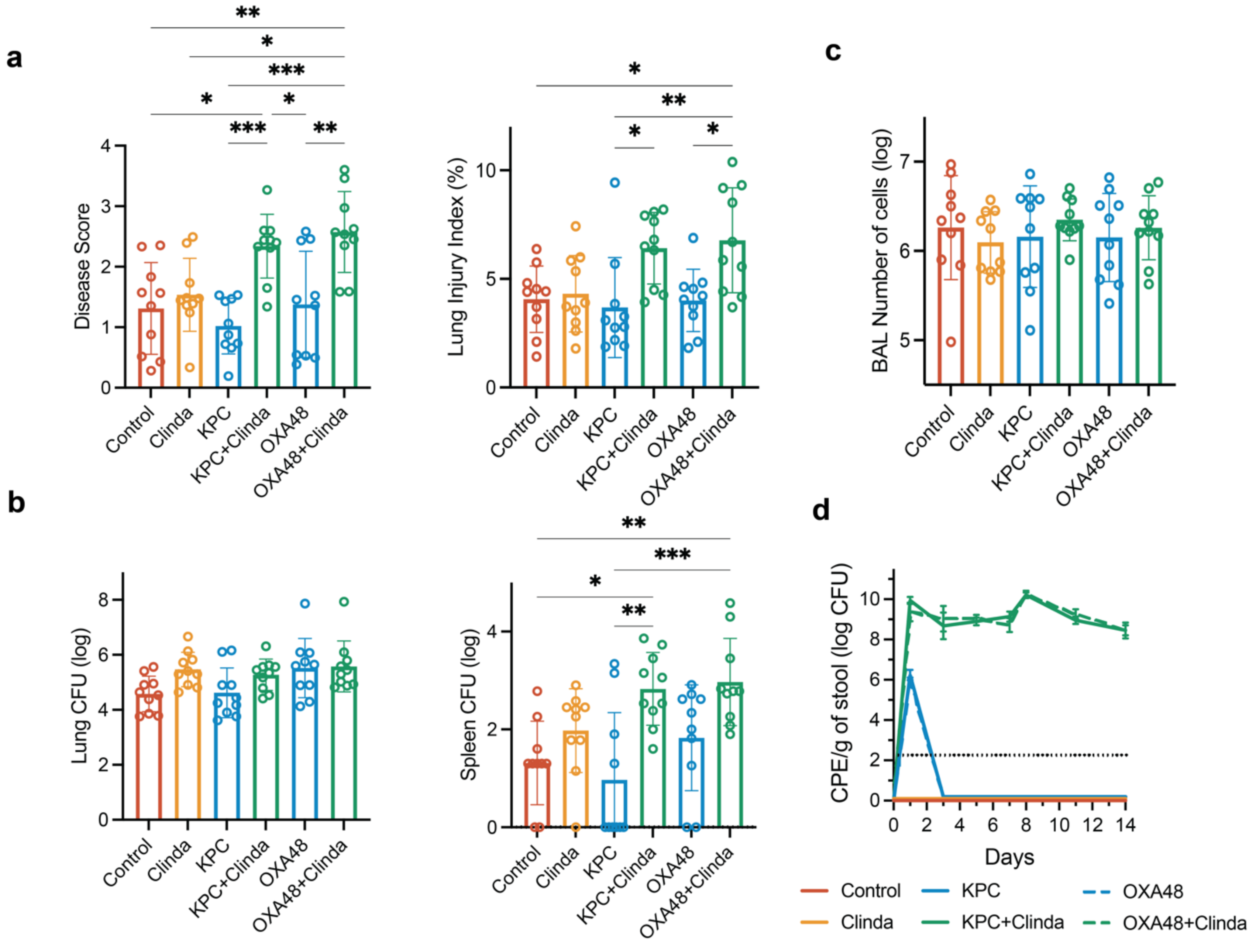

Supplementary Figure 1. Gut colonisation by other strains of carbapenemase-producing *Klebsiella pneumoniae* (KPC and OXA-48) also increases the severity of subsequent lung infection by *Pseudomonas aeruginosa*. **a)** Clinical disease severity score and alveolar-capillary permeability index (n=10 mice/group). **b)** Bacterial load in *P. aeruginosa* in the left lung and in the spleen (n=10 mice/group). **c)** Number of cells in the bronchoalveolar lavage fluid (n=10 mice/group). Data are presented as mean values ± SD of biologically independent samples from different mice. *: $p<0.05$; **: $p<0.01$; ***: $p<0.001$ (one-way ANOVA followed by Tukey's post-hoc tests, see the Source Data file for the exact P-values). **d)** Quantification of CPE load in stools for 14 days (n=10 mice/group).

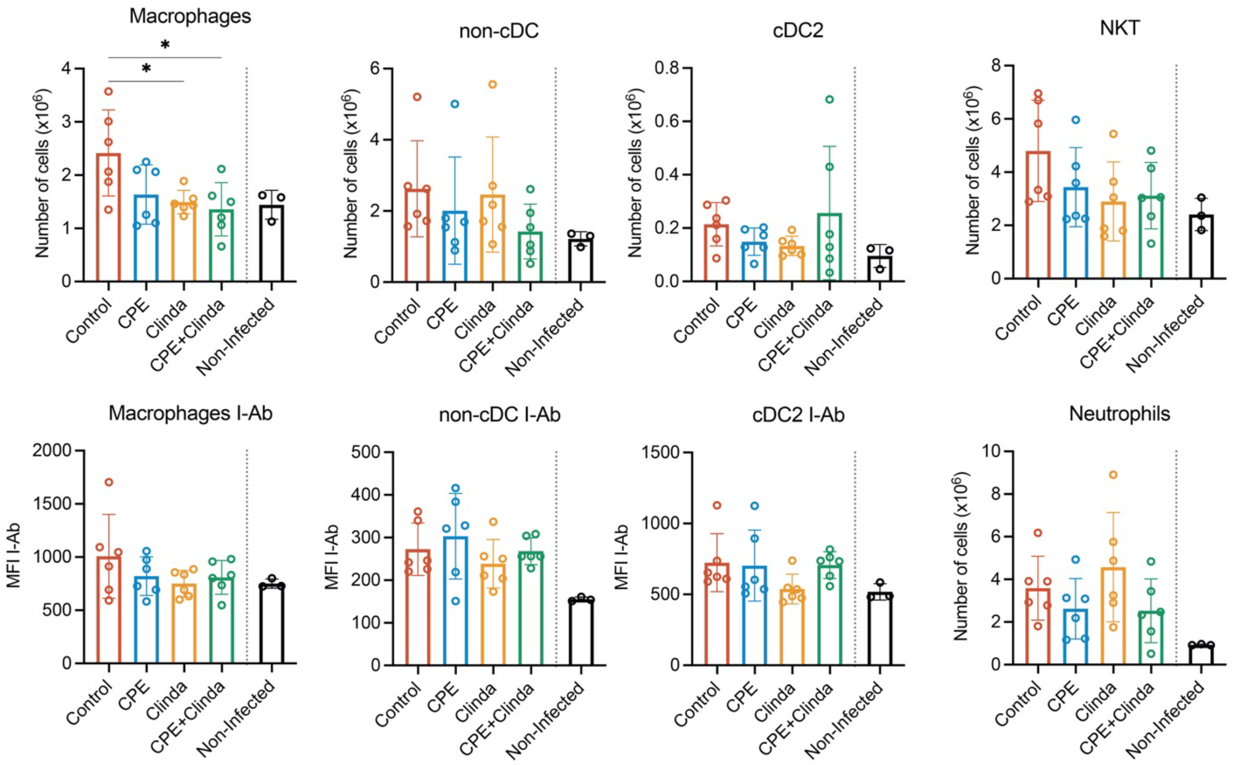

Supplementary Figure 2. Subsequent lung infection by *P. aeruginosa* effect on spleen immune cells following gut colonisation by carbapenemase-producing *Enterobacterales* (CPE). Total numbers and activation of recruited cells in the spleen: antigen-presenting cells (alveolar macrophages and conventional dendritic cells), Natural Killer T cells, neutrophils (n=6 mice/group for infected mice, n=3 for non-infected mice). Non-infected mice correspond to control mice with nasal instillation of 50 µL of PBS instead of *P. aeruginosa*. MFI: median fluorescence intensity. Data are presented as mean values ± SD of biologically independent samples from different mice. *: $p<0.05$ (one-way ANOVA followed by Tukey's post-hoc tests, see the Source Data file for the exact P-values).

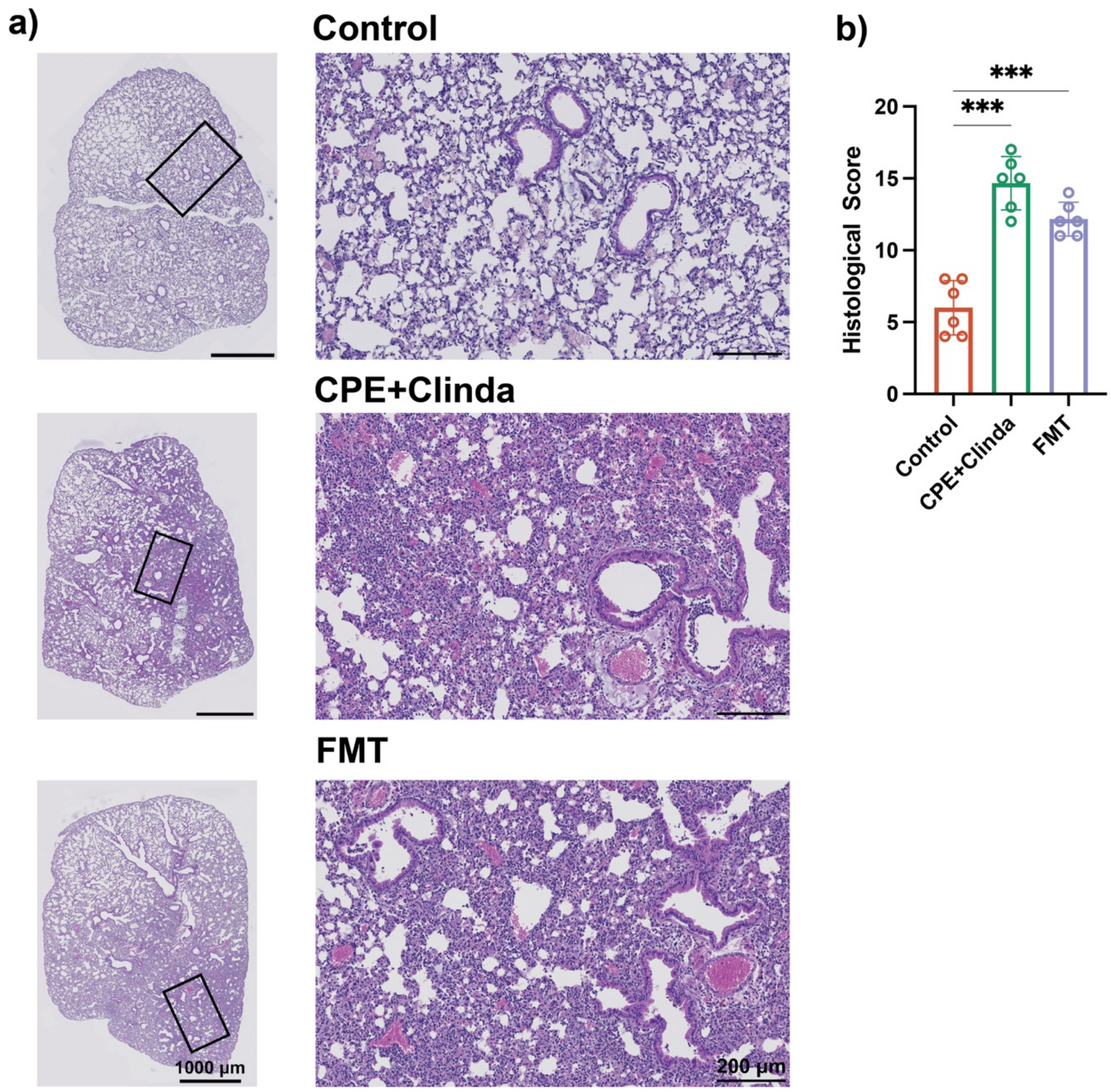

Supplementary Figure 3. Effect of faecal microbiota transplantation (FMT) in mice colonised by carbapenemase-producing *Enterobacterales* (CPE) on lung histology after *P. aeruginosa* lung infection. **a)** Representative images of lung tissue sections stained by haematoxylin and eosin (representative images out of 6 mice/group from two different experiments). Scale bars: 1000 µm (low magnification), 200 µm (high magnification). **b)** Histological score of lung infection (n=6 mice/group). Data are presented as mean values ± SD of biologically independent samples from different mice. ***: $p<0.001$ (one-way ANOVA followed by Tukey's post-hoc tests, see the Source Data file for the exact P-values).

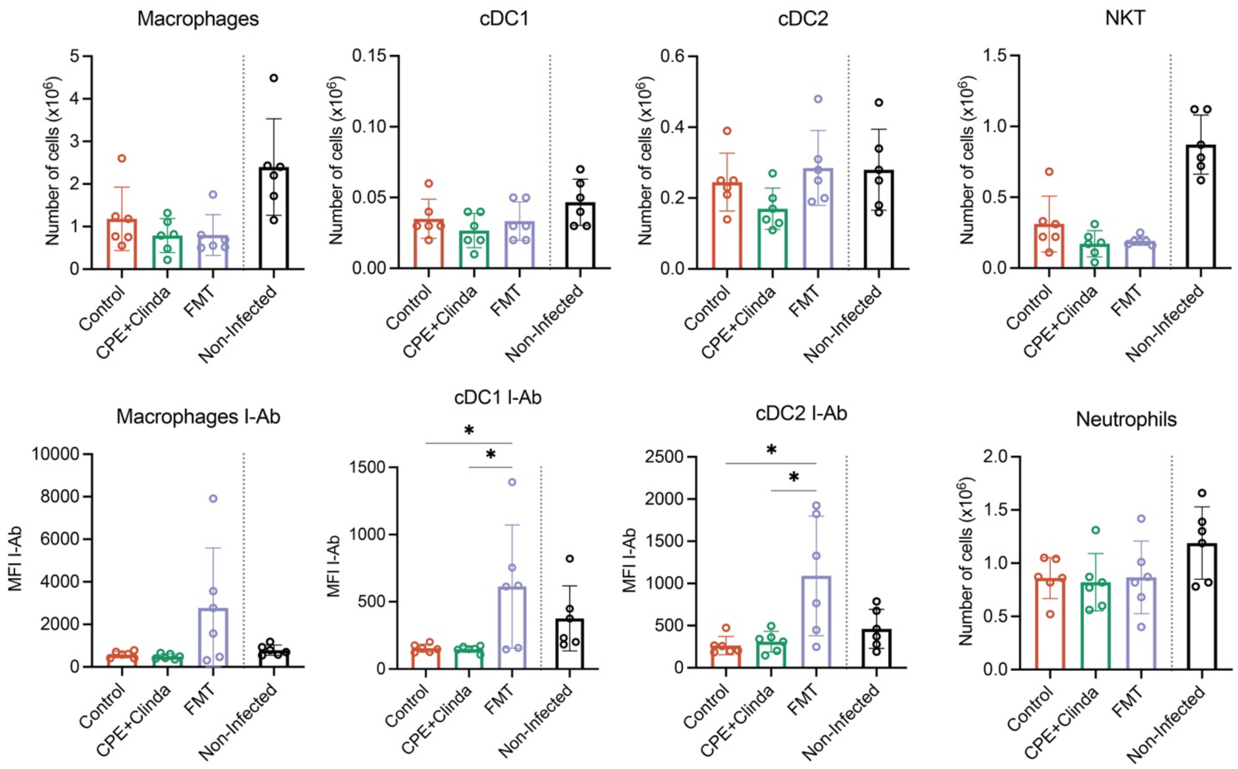

Supplementary Figure 4. Faecal microbiota transplantation (FMT) in mice colonised by carbapenemase-producing *Enterobacterales* (CPE) effect on spleen immune cells after lung infection by *P. aeruginosa*. Total numbers and activation of recruited cells in the spleen: antigen-presenting cells (alveolar macrophages and conventional dendritic cells), Natural Killer T cells, neutrophils (n=5 mice/group). Non-infected mice correspond to control mice with nasal instillation of 50 µL of PBS instead of *P. aeruginosa*. MFI: median fluorescence intensity. Data are presented as mean values ± SD of biologically independent samples from different mice. *: $p<0.05$ (one-way ANOVA followed by Tukey's post-hoc tests, see the Source Data file for the exact P-values).

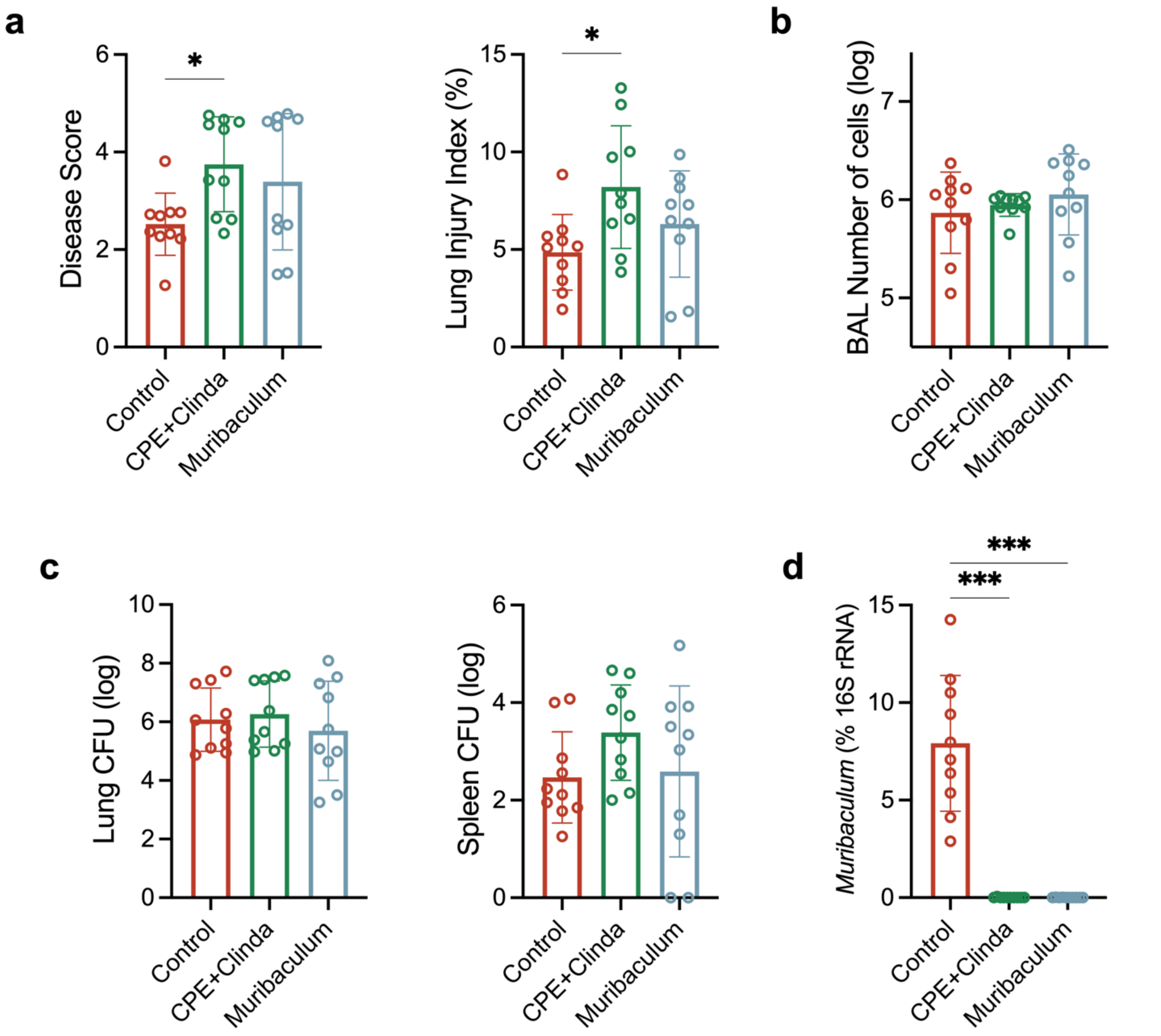

Supplementary Figure 5. Supplementation in *Muribaculum intestinale* alone does not lead to stable colonization. Mice colonised by CPE received daily orogastric gavages of *Muribaculum intestinale* DSM 28989 for the three days preceding infection by *Pseudomonas aeruginosa*. **a)** Clinical disease severity score and alveolar-capillary permeability index (n=10 mice/group). **b)** Bacterial load in *P. aeruginosa* in the left lung and in the spleen (n=10 mice/group). **c)** Number of cells in the bronchoalveolar lavage fluid (n=10 mice/group). **d)** Relative abundance in *Muribaculum* sp. assessed by qPCR at Day 14 (n=10 mice/group). Data are presented as mean values ± SD of biologically independent samples from different mice. *: p<0.05; **: p<0.01; ***: p<0.001 (one-way ANOVA followed by Tukey's post-hoc tests, see the Source Data file for the exact P-values).

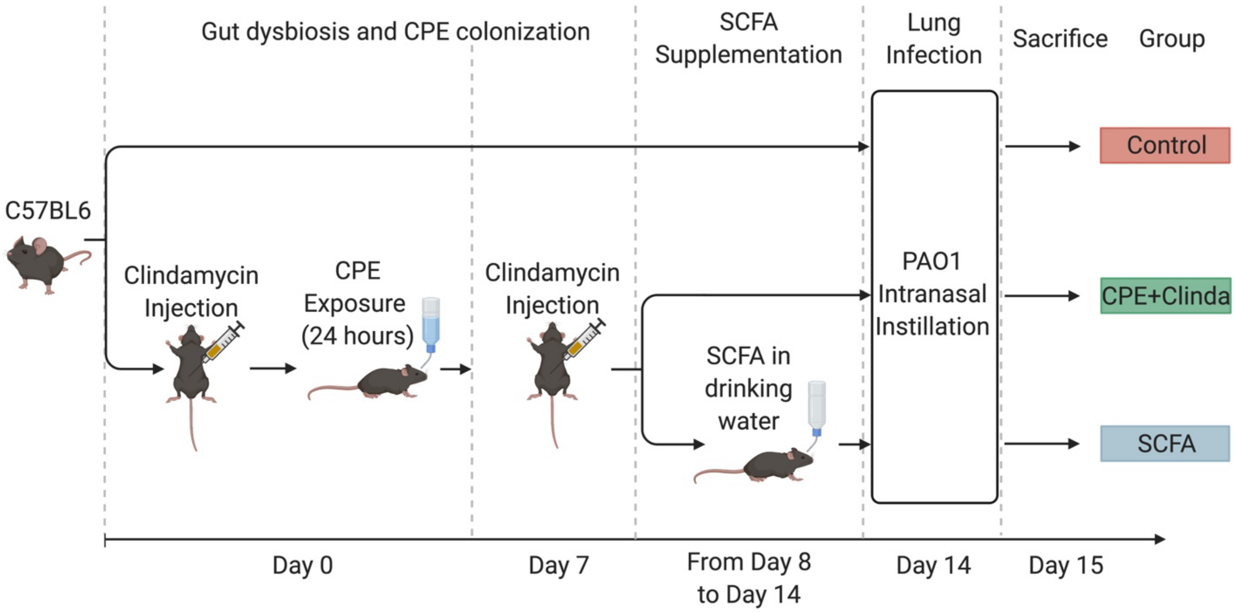

Supplementary Figure 6. Short-chain fatty acids (SCFA) supplementation: experimental plan and groups (created with BioRender.com).

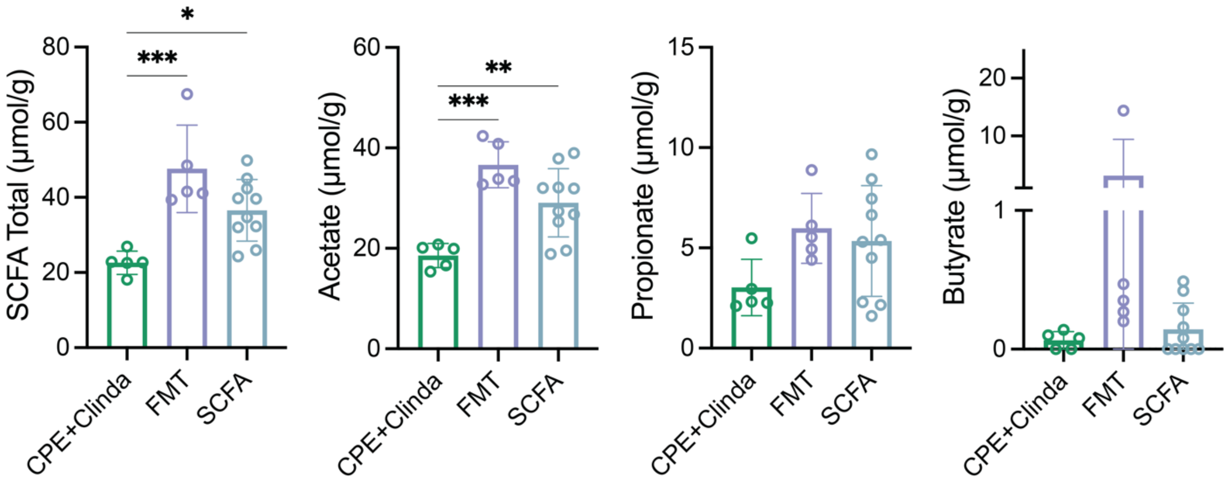

Supplementary Figure 7. Caecum concentration in short-chain fatty acids (SCFA) is increased after supplementation by a blend of SCFA for mice colonised by CPE. Quantification of SCFA in caecum lumen samples (n=5 mice/group for CPE+Clinda and FMT, n=10 mice/group for SCFA) at day 14. Data are presented as mean values ± SD of biologically independent samples from different mice. *: p<0.05; **: p<0.01; ***: p<0.001 (one-way ANOVA followed by Tukey's post-hoc tests, see the Source Data file for the exact P-values).

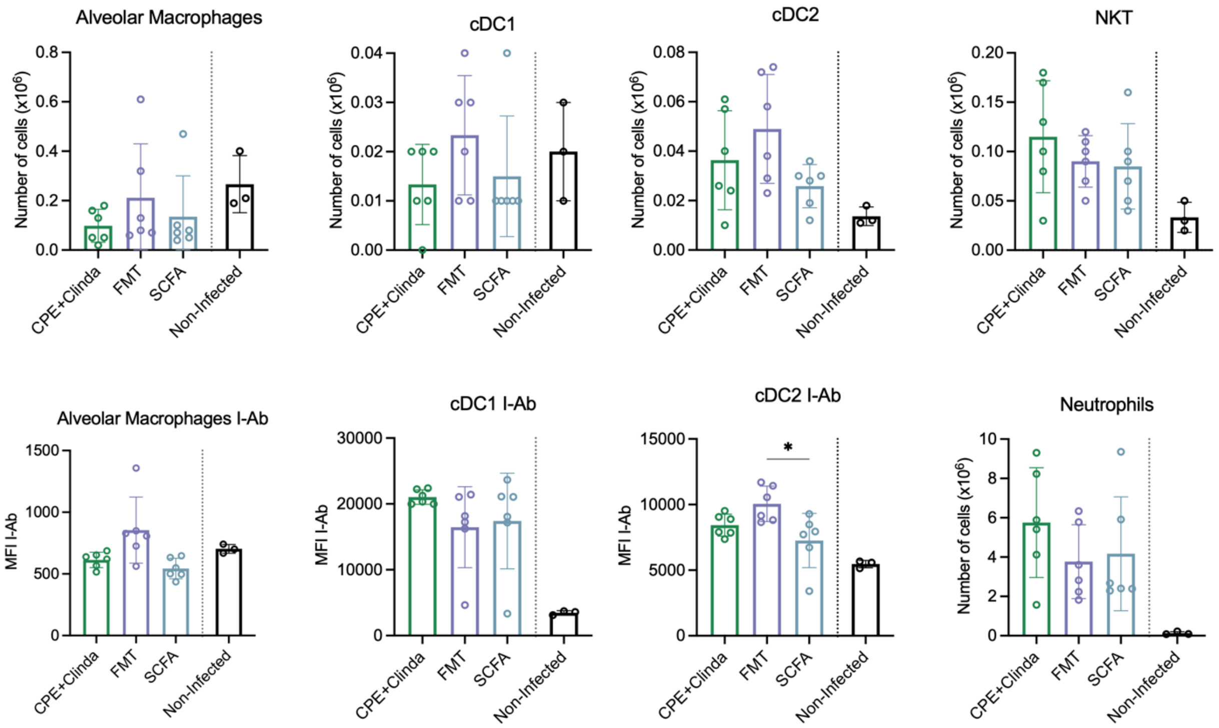

Supplementary Figure 8. SCFA supplementation presents limited effects on the recruitment of immune cells in the lungs after lung infection by *P. aeruginosa*. Total numbers and activation of recruited cells in the lung: antigen-presenting cells (alveolar macrophages and conventional dendritic cells), Natural Killer T cells, neutrophils (n=6 mice/group for infected mice, n=3 for non-infected mice). Non-infected mice correspond to control mice with nasal instillation of 50 µL of PBS instead of *P. aeruginosa*. MFI: median fluorescence intensity. Data are presented as mean values ± SD of biologically independent samples from different mice. *: p<0.05 (one-way ANOVA followed by Tukey's post-hoc tests, see the Source Data file for the exact P-values).

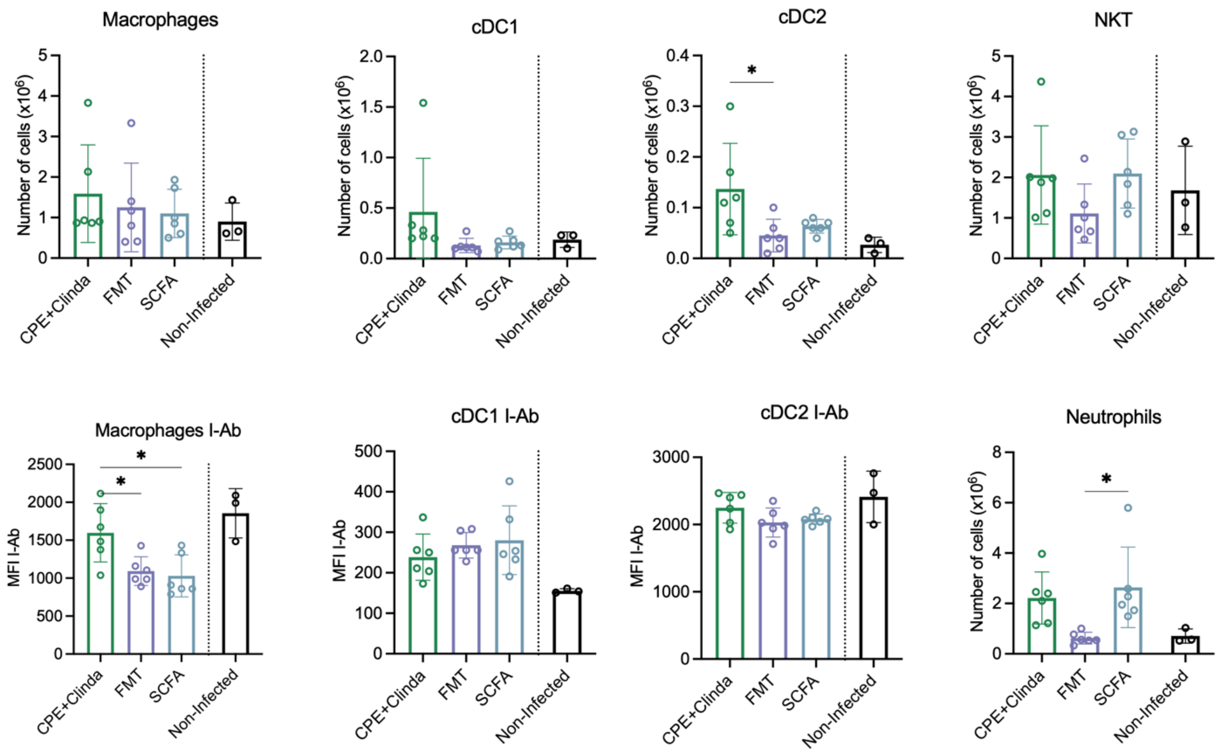

Supplementary Figure 9. SCFA supplementation presents limited effects on the recruitment of immune cells in the spleen after lung infection by *P. aeruginosa*. Total numbers and activation of recruited cells in the spleen: antigen-presenting cells (alveolar macrophages and conventional dendritic cells), Natural Killer T cells, neutrophils (n=6 mice/group for infected mice, n=3 for non-infected mice). Non-infected mice correspond to control mice with nasal instillation of 50 µL of PBS instead of *P. aeruginosa*. MFI: median fluorescence intensity. Data are presented as mean values ± SD of biologically independent samples from different mice. *: p<0.05 (one-way ANOVA followed by Tukey's post-hoc tests, see the Source Data file for the exact P-values).

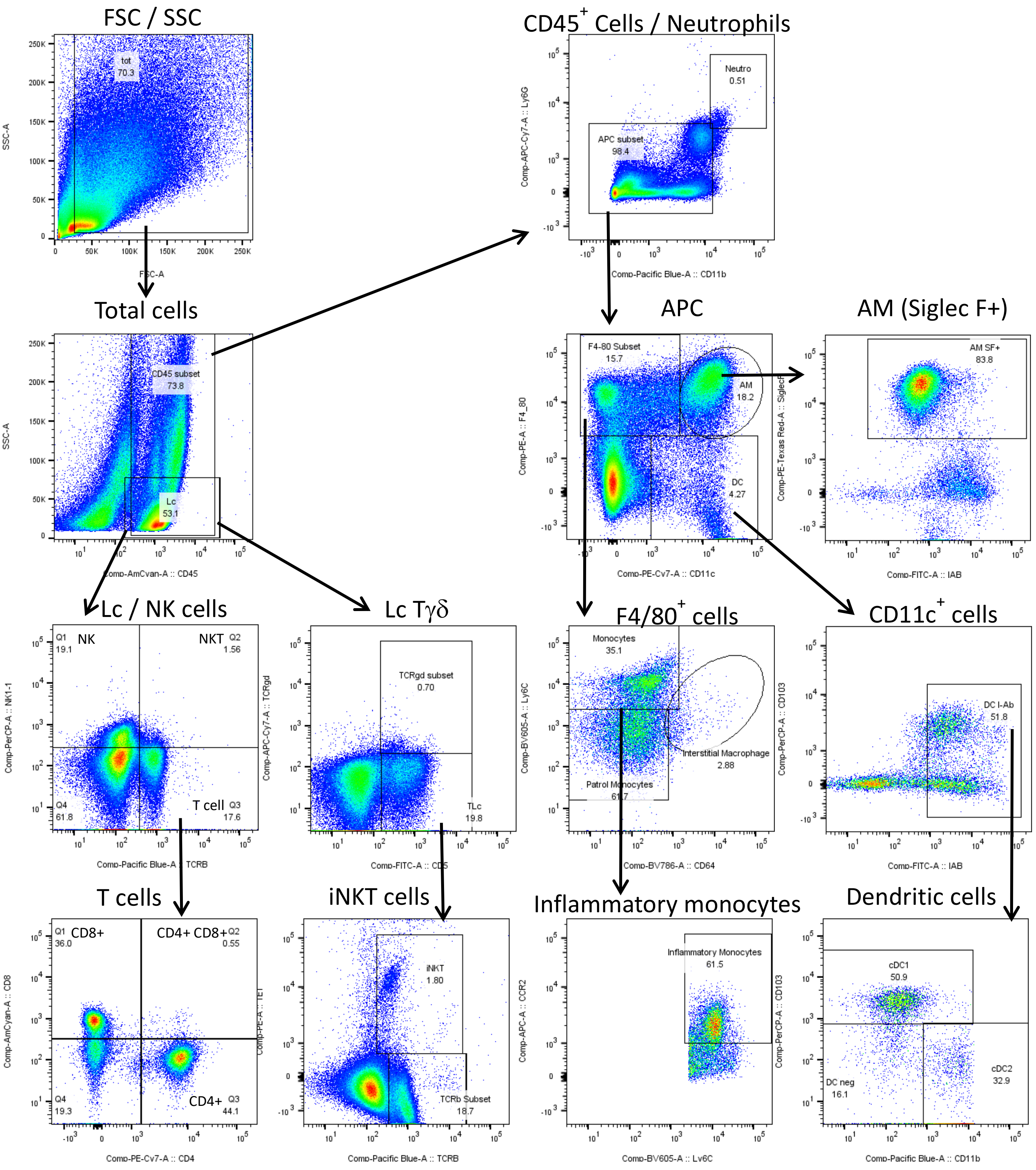
FSC / SSC
tot
70.3
SSC-A
FSC-A
Total cells
CD45 subset
73.8
Lc
53.1
Comp-AmCyan-A :: CD45
CD45+ Cells / Neutrophils
Neutro
0.51
APC subset
98.4
Comp-APC-Cy7-A :: Ly6G
Comp-Pacific Blue-A :: CD11b
APC
F4-80 Subset
15.7
AM
18.2
DC
4.27
Comp-PE-A :: F4_80
Comp-PE-Cy7-A :: CD11c
AM (Siglec F+)
AM SF+
83.8
Comp-PE-Texas Red-A :: SiglecF
Comp-FITC-A :: IAB
Lc / NK cells
Q1 NK
19.1
NKT Q2
1.56
Q4
61.8
T cell Q3
17.6
Comp-PerCP-A :: NK1-1
Comp-Pacific Blue-A :: TCRB
Lc Tγδ
TCRgd subset
0.70
TLc
19.8
Comp-APC-Cy7-A :: TCRgd
Comp-FITC-A :: CD5
F4/80+ cells
Monocytes
35.1
Interstitial Macrophage
2.88
Patrol Monocytes
61.7
Comp-BV605-A :: Ly6C
Comp-BV786-A :: CD64
CD11c+ cells
DC I-Ab
51.8
Comp-PerCP-A :: CD103
Comp-FITC-A :: IAB
T cells
Q1 CD8+
36.0
CD4+ CD8+ Q2
0.55
Q4
19.3
CD4+ Q3
44.1
Comp-AmCyan-A :: CD8
Comp-PE-Cy7-A :: CD4
iNKT cells
iNKT
1.80
TCRb Subset
18.7
Comp-PE-A :: TET
Comp-Pacific Blue-A :: TCRB
Inflammatory monocytes
Inflammatory Monocytes
61.5
Comp-APC-A :: CCR2
Comp-BV605-A :: Ly6C
Dendritic cells
cDC1
50.9
DC neg
16.1
cDC2
32.9
Comp-PerCP-A :: CD103
Comp-Pacific Blue-A :: CD11b

Supplementary Figure 10: Gating strategy for neutrophils, antigen-presenting cells (APC) and the major populations of lymphocytes (Lc) in the lung. The percentages of natural killer (NK), invariant NKT cells (iNKT) and $CD4^+$ and $CD8^+$ T Lc with a TCR$\gamma\delta$ was determined. Among APC, we identified alveolar macrophages (AM), inflammatory monocytes, patrolling monocytes, interstitial macrophages and conventional dendritic cells (cDC)1 and 2 as CD103+ and CD11b+ cells, respectively.

Supplementary Table 1. Differential abundance testing at the family level (DESeq2 with Benjamini-Hochberg False Discovery Rate) from Figure 1**.** Results are filtered based on FDR < 0.05.

Supplementary Table 2. Differential abundance testing at the genus level (DESeq2 with Benjamini-Hochberg False Discovery Rate) from Figure 1**.** Results are filtered based on FDR < 0.05.

Supplementary Table 3. Differential abundance testing at the family level (DESeq2 with Benjamini-Hochberg False Discovery Rate) from Figure 6**.** Results are filtered based on FDR < 0.05.

Supplementary Table 4. Differential abundance testing at the genus level (DESeq2 with Benjamini-Hochberg False Discovery Rate) from Figure 6**.** Results are filtered based on FDR < 0.05.

Supplementary Table 5. List of the antibodies and the ELISA kits used in this study.

Flow cytometry mAb	Target	Manufacturer	Catalog Nb	Dilution
	FITC- I-Ab	Miltenyi Biotech	130-102-168	1:200
	PE-F4/80	Miltenyi Biotech	130-102-422	1:200
	PerCP-Cy5.5 - CD103	BD Biosciences	563637	1:300
	PE-Cy7 - CD11c	BD Biosciences	558079	1:500
	APC - CCR2	Miltenyi Biotech	130-119-658	1:200
	AF700 - CD86	BD Biosciences	560581	1:150
	APC-H7- Ly6G	BD Biosciences	560600	1:500
	BV421 - CD11b	BD Biosciences	560455	1:300
	VioGreen - CD45	Miltenyi Biotech	130-110-665	1:300
	BV605 - Ly6C	Biolegend	128036	1:300
	BV786 - CD64	BD Biosciences	741024	1:500
	PE-CF594 - SiglecF	BD Biosciences	562757	1:300

	FITC - CD5	Miltenyi Biotech	130-102-574	1:300
	Tetramer mCD1d 167ms	NIH facility	30663	1:500
	PerCP-Cy5.5 - NK1.1	Miltenyi Biotech	130-103-963	1:200
	PE-Cy7 - CD4	Miltenyi Biotech	130-102-411	1:500
	APC - CD25	Miltenyi Biotech	130-102-550	1:200
	AF700 - CD69	BD Biosciences	561238	1:500
	APC-Vio770 - TCRγδ	Miltenyi Biotech	130-104-016	1:100
	VioBlue -TCRβ	Miltenyi Biotech	130-104-815	1:200
	V500 - CD8	BD Biosciences	130-109-252	1:300
	BV605 - CD45	Biolegend	103140	1:300
**ELISA kits**	**Target**	**Manufacturer**	**Catalog Nb**	
	IFN-γ ELISA kit	Invitrogen	88-7314-88	
	IL-1β Duoset	Biotechne	DY401	
	IL-6 ELISA kit	Invitrogen	88-7064-88	
	IL-17 ELISA kit	Invitrogen	88-7371-88	
	IL-22 Duoset	Biotechne	DY582	
	IL-23 ELISA kit	Invitrogen	88-7230-88	
	TNF-α ELISA kit	Invitrogen	88-7371-88	
	Defensin-β2	Abbexa	Abx254734	

Supplementary Table 6. The phenotype of the major cell populations identified in this report.

Cell population	Phenotype
Alveolar Macrophages (AM)	$CD45^+$ $F4/80^+$ $CD11c^+$ $CD64^+$ $SiglecF^+$
Neutrophils	$CD45^+$ $F4/80^-$ $CD11c^-$ $CD11b^+$ $Ly6G^+$
Dendritic cells (DC)	$CD45^+$ $F4/80^-$ $CD11c^+$ $I\text{-}Ab^+$ $CD64^-$
Inflammatory monocytes	$CD45^+$ $F4/80^+$ $CD11c^-$ $Ly6G^-$ $Ly6C^+$ $CCR2^+$
Conventional T cells	$CD45^+$ $CD5^+$ $TCR\alpha\beta^+$ $NK1.1^-$
NKT like cells	$CD45^+$ $NK1.1^+$ $TCR\alpha\beta^+$
