# Supplementary material for: Gut colonisation with multidrug-resistant Klebsiella pneumoniae worsens Pseudomonas aeruginosa lung infection
Source: Nat Commun. 2023 Jan 5;14:78. doi: 10.1038/s41467-022-35767-4 (PMC9816093; doi:10.1038/s41467-022-35767-4)
Supplement: Supplementary file 7 — Reporting Summary [file 41467_2022_35767_MOESM7_ESM.pdf]

nature portfolio

Corresponding author(s): LE GUERN Rémi

Last updated by author(s): 2022/12/21

Reporting Summary

Nature Portfolio wishes to improve the reproducibility of the work that we publish. This form provides structure for consistency and transparency in reporting. For further information on Nature Portfolio policies, see our Editorial Policies and the Editorial Policy Checklist.

Statistics

For all statistical analyses, confirm that the following items are present in the figure legend, table legend, main text, or Methods section.

n/a	Confirmed	
☐	☒	The exact sample size (n) for each experimental group/condition, given as a discrete number and unit of measurement
☐	☒	A statement on whether measurements were taken from distinct samples or whether the same sample was measured repeatedly
☐	☒	The statistical test(s) used AND whether they are one- or two-sided<br>*Only common tests should be described solely by name; describe more complex techniques in the Methods section.*
☒	☐	A description of all covariates tested
☐	☒	A description of any assumptions or corrections, such as tests of normality and adjustment for multiple comparisons
☐	☒	A full description of the statistical parameters including central tendency (e.g. means) or other basic estimates (e.g. regression coefficient) AND variation (e.g. standard deviation) or associated estimates of uncertainty (e.g. confidence intervals)
☐	☒	For null hypothesis testing, the test statistic (e.g. F, t, r) with confidence intervals, effect sizes, degrees of freedom and P value noted<br>*Give P values as exact values whenever suitable.*
☒	☐	For Bayesian analysis, information on the choice of priors and Markov chain Monte Carlo settings
☒	☐	For hierarchical and complex designs, identification of the appropriate level for tests and full reporting of outcomes
☒	☐	Estimates of effect sizes (e.g. Cohen's d, Pearson's r), indicating how they were calculated

Our web collection on statistics for biologists contains articles on many of the points above.

Software and code

Policy information about availability of computer code

Data collection	Data were collected using Excel 16.67 (Build 22110600).
Data analysis	Microbiome data were analysed using QIIME 2 2020.8. Raw sequence data were demultiplexed and quality filtered with the q2-demux plugin, followed by denoising with DADA2. Taxonomy was assigned to amplicon sequence variants (ASVs) using the q2-feature-classifier plugin against the SILVA 132 99% database. ASVs clustered in phylogenetic levels were further analysed using the phyloseq package in R software version 4.2.2 (R Core Team, Vienna, Austria) to perform α-diversity estimates and plot relative abundances. β-diversity was analysed with principal coordinate analysis (PCoA) of Bray-Curtis distances using the R package microbial. Differential abundance testing was performed using DESeq2 and applying Benjamini-Hochberg False Discovery Rate.<br>Statistical analysis was performed using GraphPad Prism 9.2 (GraphPad Software, La Jolla, CA, USA) and R 4.2.2 (R Core Team, Vienna, Austria).<br>Flow cytometry data were analysed using FlowJo™ software v7.6.5.<br>Histologic slides were numerised using Axio Scan Z1 (Zeiss) and analysed with ZEN 3.3 software.

For manuscripts utilizing custom algorithms or software that are central to the research but not yet described in published literature, software must be made available to editors and reviewers. We strongly encourage code deposition in a community repository (e.g. GitHub). See the Nature Portfolio guidelines for submitting code & software for further information.

Data

Policy information about availability of data

All manuscripts must include a data availability statement. This statement should provide the following information, where applicable:

- Accession codes, unique identifiers, or web links for publicly available datasets
- A description of any restrictions on data availability
- For clinical datasets or third party data, please ensure that the statement adheres to our policy

Microbiome sequencing data have been deposited to Sequence Read Archive (BioProject PRJNA780243), available on the following access link: https://www.ncbi.nlm.nih.gov/bioproject/?term=PRJNA780243
Source data are provided with this paper.

Field-specific reporting

Please select the one below that is the best fit for your research. If you are not sure, read the appropriate sections before making your selection.

[x] Life sciences [ ] Behavioural & social sciences [ ] Ecological, evolutionary & environmental sciences

For a reference copy of the document with all sections, see nature.com/documents/nr-reporting-summary-flat.pdf

Life sciences study design

All studies must disclose on these points even when the disclosure is negative.

Sample size	For animal research, we used power analysis and sample size calculations (α = 0.05 and (1-β) = 0.8) to ensure reproducibility according to the 3R's principles.
Data exclusions	Mice that failed to survive 18 hours after lung infection were excluded.
Replication	We state the number of mice in each experiment (usually 10-15 mice per group). Overall, our findings were reliably reproduced, including necessary controls. For histological analyses, we selected the most representative images based on the histological score.
Randomization	Mice were all male and from the same age (6 to 8 weeks) for all animal experiments. Animals were randomly assigned to groups before experimentation.
Blinding	Mice experiments were not performed in a blinded fashion. Mice exposed to multidrug-resistant bacteria or infected by Pseudomonas aeruginosa had to be properly labeled for laboratory safety. However, histology slides were scored in a blinded fashion.

Reporting for specific materials, systems and methods

We require information from authors about some types of materials, experimental systems and methods used in many studies. Here, indicate whether each material, system or method listed is relevant to your study. If you are not sure if a list item applies to your research, read the appropriate section before selecting a response.

Materials & experimental systems

n/a	Involved in the study	
[ ]	[x]	Antibodies
[x]	[ ]	Eukaryotic cell lines
[x]	[ ]	Palaeontology and archaeology
[ ]	[x]	Animals and other organisms
[x]	[ ]	Human research participants
[x]	[ ]	Clinical data
[x]	[ ]	Dual use research of concern

Methods

n/a	Involved in the study	
[x]	[ ]	ChIP-seq
[x]	[ ]	Flow cytometry
[x]	[ ]	MRI-based neuroimaging

Antibodies

Antibodies used

Flow cytometry mAb Target Manufacturer Catalog Nb
FITC- I-Ab, Miltenyi Biotech, 130-102-168,1:200
PE-F4/80, Miltenyi Biotech, 130-102-422, 1:200
PerCP-Cy5.5- CD103, BD Biosciences, 563637, 1:300
PE-Cy7 - CD11c, BD Biosciences, 558079, 1:500
APC - CCR2, Miltenyi Biotech, 130-119-658 ,1:200

AF700 - CD86, BD Biosciences, 560581, 1:150
APC-H7- Ly6G, BD Biosciences, 560600, 1:500
V450 - CD11b, BD Biosciences, 560455 , 1:300
VioGreen - CD45, Miltenyi Biotech, 130-110-665, 1:300
BV605 - Ly6C, Biolegend, 128036,1:300
BV786 - CD64, BD Biosciences, 741024, 1:500
PE-CF594 – SiglecF, BD Biosciences, 562757, 1:300
FITC - CD5, Miltenyi Biotech, 130-102-574, 1:300
Tetramer mCD1d 167ms, NIH facility, 30663, 1:500
PerCP-Cy5.5 - NK1.1, Miltenyi Biotech, 130-103-963, 1:200
PE-Cy7 - CD4, Miltenyi Biotech, 130-102-411,1:500
APC - CD25, Miltenyi Biotech, 130-102-550, 1:200
AF700 - CD69, BD Biosciences, 561238, 1:500
APC-Vio770 – TCRγδ, Miltenyi Biotech, 130-104-016 , 1:100
VioBlue –TCRβ, Miltenyi Biotech, 130-104-815, 1:200
V500 - CD8, BD Biosciences, 130-109-252, 1:300
BV605 - CD45, Biolegend, 103140, 1:300

ELISA kits Target, Manufacturer ,Catalog Nb
IFN-g ELISA kit, Invitrogen, 88-7314-88,
IL-1β Duoset, Biotechne, DY401
IL-6 ELISA kit, Invitrogen, 88-7064-88,
IL-17 ELISA kit, Invitrogen, 88-7371-88 ,
IL-22 Duoset, Biotechne, DY582
IL-23 ELISA kit, Invitrogen, 88-7230-88
TNF-α ELISA kit, Invitrogen, 88-7371-88

Validation

All the antibodies listed above were validated by their respective manufacturers:

- BD Biosciences
"Specificity of antibodies
BD Biosciences not only develops its own antibodies but also collaborates with research scientists around the world to license their antibodies. We provide accessibility to the flow cytometry community by conjugating antibodies to a broad portfolio of high-performing dyes, including our vastly popular portfolio of BD Horizon Brilliant™ Dyes. A world-class team of research scientists helps ensure that these reagents work reliably and consistently for flow cytometry applications. The specificity is confirmed by using multiple applications that may include a combination of flow cytometry, immunofluorescence, immunohistochemistry or western blot to test a combination of primary cells, cell lines or transfectant models.
All flow cytometry reagents are titrated on the relevant positive or negative cells. To save time and cell samples for researchers, pre-titrated test size reagents are bottled at an optimal concentration, with the best signal-to-noise ratio on relevant models. You can look up the Certificate of Analysis and the concentration of test-size human reagents from specific lots via the Concentration Lookup: https://www.bdbiosciences.com/en-fr/support/product-support/concentration-lookup page or BD Regulatory Documents https://www.bdbiosciences.com/en-fr/support/documents
Technical data sheets provide data generated on the relevant primary model at this optimal concentration based on a titration curve. QC data on any lot of reagent can be requested through ResearchApplications@bd.com.
Quality control. Our dedication to rigorous testing and high-quality control standards means that you can work on your research with the utmost confidence. All BD reagent facilities, including our California Design Center at San Diego, our manufacturing facility located at Tatabanya, Hungary and our California instrument facility (Manufacturing and Design Centers) at San Jose, are approved and registered to the internationally defined ISO 9001 standard. Once our research and development (R&D) team completes evaluation of a new product, the developed process is transferred to our manufacturing teams, including Quality Control. Our manufacturing process adheres to standard operating procedures (SOPs) and guidelines, conforming to ISO requirements, and is strictly followed, helping ensure that reagent builds provide consistent results to help give you assurance of experimental success and confidence in your research. Quality control testing of new, manufactured lots are performed side-by-side with a previously accepted lot as a control, helping to serve as a reference for comparison and assuring that performance of the new lot is both reliable and consistent. Our strict adherence to these guidelines helps ensure that different lots of conjugated reagents are performing consistently.
Lot-to-lot consistency. We understand the criticality of lot-to-lot consistency in helping you obtain experimental success and confidence in your research. We strive to ensure different production batches are consistent regardless of type of antigen or fluorochrome through exhaustive testing and strict adherence to quality control standards. Testing with prior batches as reference helps you obtain consistent results with the new batch relative to the previous batches.
https://www.bdbiosciences.com/en-us/products/reagents/flow-cytometry-reagents/research-reagents/quality-and-reproducibility"

- Miltenyi Biotech
"With the introduction of recombinant antibodies in 2012, we made a significant investment into improving the quality and consistency of our antibodies. The standardized antibody production process, starting from a defined DNA sequence, and the nature of recombinant antibodies ensure high purity and lot-to-lot consistency.
In addition, recombinant antibodies do not display any undesired mixtures of heavy and light immunoglobulin chains, which is often the case with conventional hybridoma-derived antibodies (PMID: 29485921). Furthermore, our REAfinity™ Recombinant Antibodies have a mutated Fc region that abolishes any binding to Fc# receptors, resulting in a background-free analysis. These advantages make REAfinity Recombinant Antibodies ideal tools for improving experimental reproducibility.
In 2020, we began the further step of providing antibody validation data directly on our product pages. We do this in order to make it even easier for our customers to choose the antibodies that best match their needs, and to decrease the validation efforts required

on their researcher side. With well over 10,000 antibodies in our portfolio, this is an ongoing project and information is updated regularly, so please do check back from time to time. In addition, below you can find some insights on how we conduct our antibody validation process.
https://www.miltenyibiotec.com/US-en/products/macs-antibodies/antibody-validation.html"

- BioLegend
"All of our products undergo industry-leading rigorous quality control (QC) testing to ensure the highest level of performance and reproducible results. Each lot is compared to an internally established “gold standard” to maintain lot-to-lot consistency. We also conduct wide-scale stability studies to guarantee an accurate shelf-life for our products.
Additionally, we test the majority of our products on endogenous cells rather than transfected or immortal cells that may overexpress the analyte. We assess our reagents with samples and protocols that reflect our customers' experience. Our willingness to monitor the quality of our reagents extends beyond our lab and into yours.
• Specificity testing of 1-3 target cell types with either single- or multi-color analysis (including positive and negative cell types).
• Once specificity is confirmed, each new lot must perform with similar intensity to the in-date reference lot. Brightness (MFI) is evaluated from both positive and negative populations.
• Each lot product is validated by QC testing with a series of titration dilutions.
https://www.biolegend.com/fr-fr/quality/quality-control"

Animals and other organisms

Policy information about studies involving animals; ARRIVE guidelines recommended for reporting animal research

Laboratory animals	Male C57/Bl6JRj mice aged 6 to 8 weeks were purchased from Janvier Labs and housed under specific pathogen-free conditions. Mice had free access to a standard laboratory food diet and water, and were housed under a 12 h light-dark cycle, 50–70% humidity and 20–24°C temperature.
Wild animals	The study did not involve wild animals.
Field-collected samples	The study did not involve samples collected from the field.
Ethics oversight	The animal procedure followed in this study was in accordance with the French Guidelines for the Care and Use of Laboratory Animals and the European Union guidelines. The current project has been approved by the National Institutional Animal Care and Use Committee (CEEA 75) and received the authorisation number APAFIS #7166.

Note that full information on the approval of the study protocol must also be provided in the manuscript.
